# Rapid Radiographic Improvement in Large Multilobulated Shoulder Calcific Tendinitis After Ultrasound-Guided Prolotherapy and Adjunctive Nutritional and Metabolic Support: A Case Report

**DOI:** 10.7759/cureus.106864

**Published:** 2026-04-11

**Authors:** Yonghyun Yoon, Jaeyoung Lee, Jaewoo Lim, Ji Hyo Hwang, Teinny Suryadi, Anwar Suhaimi, King Hei Stanley Lam

**Affiliations:** 1 Orthopedics, International Academy of Musculoskeletal Medicine, Hongkong, HKG; 2 Orthopedics, International Academy of Regenerative Medicine, Incheon, KOR; 3 Orthopedics, MSKUS, Vista, USA; 4 Orthopedic Surgery, Hallym University Kangnam Sacred Heart Hospital, Seoul, KOR; 5 Orthopedic Surgery, Incheon Terminal Orthopedic Surgery Clinic, Incheon, KOR; 6 Orthopedics, Incheon Terminal Orthopedic Surgery Clinic, Incheon, KOR; 7 Physical Medicine and Rehabilitation, Synergy Clinic, Jakarta, IDN; 8 Physical Medicine and Rehabilitation, Hermina Hospital Podomoro, Jakarta, IDN; 9 Rehabilitation Medicine, University Malaya Medical Centre, Kuala Lumpur, MYS; 10 Rehabilitation Medicine, University Malaya, Kuala Lumpur, MYS; 11 Faculty of Medicine, The Chinese University of Hong Kong, New Territories, HKG; 12 Faculty of Medicine, The University of Hong Kong, Hong Kong, HKG; 13 The Board of Clinical Research, The Hong Kong Institute of Musculoskeletal Medicine, Kowloon, HKG

**Keywords:** calcific tendinitis, nutritional and metabolic support, prolotherapy, shoulder, ultrasound-guided injection

## Abstract

Calcific tendinitis of the shoulder is a common disorder characterized by calcium hydroxyapatite deposition within or near the rotator cuff tendons. Although the condition is often self-limited, large multilobulated calcific deposits may be less responsive to conservative treatment and can prompt consideration of more invasive procedures. We report a case of a 49-year-old man who presented with acute severe right shoulder pain, sleep disturbance, and marked functional limitation. At presentation, pain was 10/10 on the visual analog scale, and the shoulder range of motion could not be formally assessed because of severe pain. Plain radiographs demonstrated a large multilobulated calcific deposit measuring approximately 3 cm in the subacromial region, with a smaller adjacent calcification. Ultrasound-guided prolotherapy was performed using a solution of 10% dextrose with a final lidocaine concentration of 0.2%, with 5 mL injected into the glenohumeral joint and 5 mL into the subacromial space. Adjunctive management included hydration, physical therapy, activity modification, and adjunctive nutritional and metabolic support consisting of vitamin C, vitamin D, magnesium, methylsulfonylmethane, and L-arginine. At two weeks, pain improved to 2/10; passive range of motion was nearly full; active range of motion was full, with only mild pain at terminal motion; and follow-up radiographs demonstrated near-complete resorption of the calcific deposit. Although causality cannot be established from a single case, this report suggests that selected patients with calcific tendinitis may experience rapid improvement with a multimodal nonoperative strategy incorporating adjunctive nutritional and metabolic support before escalation to invasive treatment.

## Introduction

Calcific tendinitis of the shoulder is a common musculoskeletal disorder characterized by calcium hydroxyapatite deposition within or adjacent to the rotator cuff tendons [1-3]. Clinical presentation varies according to disease stage, and patients in the resorptive phase often present with abrupt severe pain and marked functional limitation [1,4]. Conservative management typically includes analgesics, physical therapy, subacromial injection, ultrasound-guided needling/lavage, extracorporeal shock wave therapy, and, in refractory cases, surgery [2,3]. Large deposits, particularly those with a multilobulated morphology, are often considered less responsive to conservative treatment and may prompt consideration of more invasive procedures [3-5].

Although local treatment strategies have been widely discussed, potentially modifiable systemic contributors are less frequently emphasized in routine practice [2,3]. Nutritional and metabolic support may represent an adjunctive consideration in selected patients before escalating to invasive intervention. We report a case of large multilobulated calcific tendinitis of the shoulder that showed rapid radiographic improvement following ultrasound-guided prolotherapy combined with adjunctive nutritional and metabolic support.

## Case presentation

A 49-year-old man presented with severe right shoulder pain and functional limitation. His medical history was notable only for gout, with no relevant family history, recent trauma, unusual physical exertion, or corticosteroid exposure. He had worked in an office setting for many years and did not report any specific sports or hobby-related overuse. The pain had acutely worsened one day prior to presentation and became severe enough to interfere with sleep and routine daily activities. At presentation, the pain intensity was 10/10 on the visual analog scale (VAS), and the shoulder range of motion could not be formally assessed because of severe pain.

**Figure 1:**
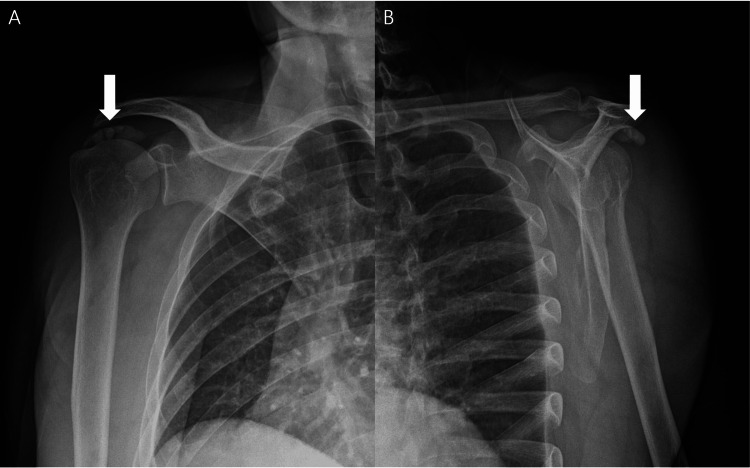
Large multilobulated calcific tendinitis of the right shoulder. (A) Anteroposterior radiograph of the right shoulder obtained at presentation shows a large, multilobulated calcific deposit measuring approximately 3 cm in the subacromial region (arrow), with a smaller adjacent calcification. (B) Scapular Y-view radiograph obtained at the same visit confirms the location of the calcific deposits in the subacromial region (arrow).

Plain radiographs of the right shoulder demonstrated a large multilobulated calcific deposit measuring approximately 3 cm in the subacromial region, accompanied by a smaller adjacent calcification (Figures 1A, 1B). Cervical spine radiographs showed degenerative changes, but no findings sufficient to explain the severity of the shoulder symptoms.

The patient underwent ultrasound-guided prolotherapy using a solution of 10% dextrose with a final lidocaine concentration of 0.2%. Under ultrasound guidance, 5 mL was injected into the glenohumeral joint and an additional 5 mL into the subacromial space. In addition, adjunctive nutritional and metabolic support was initiated, including increased oral hydration, with a recommendation of approximately 2 L of water per day based on general hydration guidelines and daily supplementation with vitamin C 10 g, vitamin D 5,000 IU, magnesium 500 mg, methylsulfonylmethane (MSM) 2,000 mg, and L-arginine 2,000 mg [6]. He was also advised to undergo physical therapy three times weekly and to temporarily avoid overhead activity. Nonsteroidal anti-inflammatory drugs were not emphasized during the treatment course.

**Figure 2:**
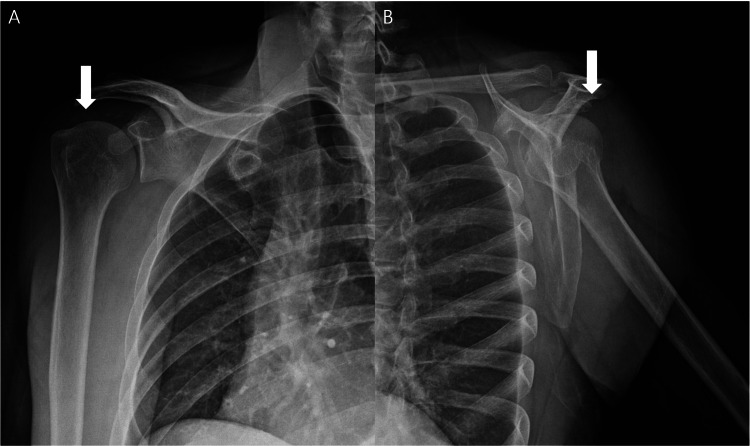
Near-complete radiographic resorption two weeks later. (A) Follow-up anteroposterior radiograph obtained two weeks after presentation shows near-complete resorption of the calcific deposit (arrow). (B) Follow-up scapular Y-view radiograph demonstrates marked reduction of the calcification (arrow).

At the two-week follow-up, the patient reported marked clinical improvement, with pain decreasing from VAS 10/10 to 2/10. Passive shoulder range of motion was nearly full, and active range of motion had returned to full in all planes, although mild residual pain persisted at terminal motion. Follow-up radiographs obtained at the same visit demonstrated near-complete resorption of the previously identified calcific deposits (Figures 2A, 2B).

## Discussion

Calcific tendinitis of the shoulder is generally understood as a self-limited condition that progresses through formative, resting, and resorptive phases [1,2,4]. Although spontaneous improvement can occur, radiographic resolution is typically expected over a period of months rather than weeks [1,2,7]. Larger deposits, particularly those with multilobulated morphology, may be associated with poorer response to conservative management and may prompt consideration of more invasive options such as ultrasound-guided needling, extracorporeal shock wave therapy, or surgery [8,9]. In the present case, the patient had a large multilobulated calcific deposit measuring approximately 3 cm, which may be less favorable.

This case is notable for the combination of marked clinical and radiographic improvement within only two weeks. Pain improved from a visual analog scale (VAS) score of 10/10 at presentation to 2/10 at follow-up, while shoulder motion improved from being unassessable because of severe pain to nearly full passive motion and full active motion with only mild residual pain at terminal range. Follow-up radiographs obtained at the same two-week visit demonstrated near-complete resorption of the calcific deposits. Given the initial size and multilobulated appearance of the lesion, the rapidity of improvement appears disproportionate to the expected natural history.

The treatment strategy in this case was multimodal. The patient underwent ultrasound-guided prolotherapy with increased oral hydration, temporary activity modification, physical therapy, and adjunctive nutritional and metabolic support consisting of vitamin C, vitamin D, magnesium, methylsulfonylmethane (MSM), and L-arginine [10-14]. Because these measures were introduced together, the relative contribution of each component cannot be determined. Accordingly, this report should not be interpreted as evidence that any single intervention directly caused calcific resorption. Rather, it documents a favorable clinical course temporally associated with a combined local treatment approach and adjunctive nutritional and metabolic support. This broader rationale is supported by prior clinical studies showing that adjunctive nutritional supplementation, when combined with conventional treatment such as extracorporeal shock wave therapy, may improve pain and functional outcomes in tendinopathy, including insertional Achilles tendinopathy [15,16]. Although those data were not specific to calcific tendinitis, the present case followed a similar rationale, incorporating adjunctive nutritional and metabolic support into a multimodal conservative approach for a calcific tendon disorder.

Previous case reports have described successful use of ultrasound-guided prolotherapy in calcification-related pain syndromes outside the shoulder, such as A1 pulley calcification causing trigger finger and sacrospinous ligament calcification associated with deep gluteal syndrome [17,18]. In this context, the present case expands the clinical spectrum in which prolotherapy may be considered as part of multimodal conservative management.

One of the clinically relevant points raised by this study is that management of calcific tendinitis often becomes focused on procedural escalation once symptoms are severe or radiographs show a large deposit. However, the possibility that local recovery may also be influenced by broader biologic conditions should not be overlooked. Potentially modifiable nutritional and metabolic factors are less frequently emphasized in routine treatment discussions, despite the fact that tendon healing and inflammatory resolution are not purely local processes. In this context, adjunctive nutritional and metabolic support may deserve consideration as an adjunctive component of nonoperative care before proceeding to more invasive interventions in selected patients.

This study has several important limitations. First, it is based on a single case, which limits generalizability and precludes definitive conclusions. Second, although pain scores and range of motion were documented, standardized functional outcome measures and biochemical assessments were not systematically applied. Third, the multimodal treatment approach prevents the determination of the individual contribution of each intervention, and causality cannot be established. In addition, it is possible that the patient presented during an active resorptive phase, and that part of the observed improvement reflects the natural course of the disease. The relatively short follow-up period further limits assessment of long-term outcomes.

Therefore, this case should be considered hypothesis-generating rather than confirmatory. Nevertheless, the unusually rapid radiographic resolution of a large multilobulated calcification suggests that further investigation of combined nonoperative strategies, including nutritional and metabolic support, may be warranted in selected patients.

## Conclusions

This case describes rapid clinical and radiographic improvement of a large multilobulated calcific tendinitis of the shoulder following a multimodal nonoperative approach consisting of ultrasound-guided prolotherapy, physical therapy, activity modification, hydration, and adjunctive nutritional and metabolic support. Although causality cannot be established from a single case, the marked reduction in pain, recovery of shoulder motion, and near-complete radiographic resorption within two weeks suggest that selected patients may improve without immediate escalation to invasive treatment. Before proceeding to more aggressive procedures, clinicians may consider whether potentially modifiable nutritional and metabolic factors have been adequately addressed as part of a comprehensive conservative treatment strategy.
